# Harnessing nutrients and natural products for sustainable drug development against aging

**DOI:** 10.3389/fphar.2025.1579266

**Published:** 2025-04-28

**Authors:** Fuan Ding, Ying Yu, Yan Zhang, Shibo Wei, Jung Ho Han, Zhuo Li, Hong-Bo Jiang, Dongryeol Ryu, Wonyoung Park, Ki-Tae Ha, Li Geng

**Affiliations:** ^1^ Department of Vascular Surgery, The Second Hospital of Jilin University, Changchun, China; ^2^ Department of Surgery, Changchun University of Chinese Medicine, Changchun, China; ^3^ Department of Biomedical Science and Engineering, Gwangju Institute of Science and Technology, Gwangju, Republic of Korea; ^4^ Korean Medicine Application Center, Korea Institute of Oriental Medicine, Daegu, Republic of Korea; ^5^ Department of Nephrology, Shandong Provincial Hospital Affiliated to Shandong First Medical University, Jinan, Shandong, China; ^6^ Department of Dermatology, Qingdao Women and Children’s Hospital, Qingdao University, Qingdao, Shandong, China; ^7^ Department of Korean Medical Science, School of Korean Medicine, Pusan National University, Yangsan, Gyeongsangnam-do, Republic of Korea; ^8^ Research Institute for Korean Medicine, Pusan National University, Yangsan, Gyeongsangnam-do, Republic of Korea

**Keywords:** aging, nutrients, natural products, pharmaceuticals, healthspan

## Abstract

Developing treatments for age-related diseases requires cost-effective and efficient approaches. Nutrients and natural metabolites offer safer alternatives to synthetic drugs. Aging increases the need for solutions that protect health and repair cells. Recent studies show that nutrients and natural products reduce oxidative stress, regulate metabolism, and influence longevity-related genes. This review focuses on vitamins, minerals, antioxidants, and natural products that improve healthspan and combat aging. It also discusses challenges such as standardization, clinical validation, and regulatory approval. Finally, emerging trends, such as personalized nutrition and advanced delivery systems, highlight the potential of these metabolites for addressing aging.

## 1 Introduction

The increasing number of aged people in the world is causing more problems, especially diseases related to age (Prince et al., 2015; Jaul and Barron, 2017). This situation puts a lot of pressure on healthcare systems (Organization, 2001; Evans and Stoddart, 2017). Therefore, new treatment development is needed strongly that make life longer and improve the quality (Partridge et al., 2018). The increasing prevalence of age-related diseases, such as cardiovascular disorders and neurodegeneration, underscores the need for sustainable solutions (Collaborators G. A., 2022; Collaborators G. M. D., 2022). These solutions should focus on improving both lifespan and healthspan (López-Otín et al., 2013). To solve this issue, research on nutrients and natural products is becoming an important area (Cragg and Newman, 2013; Li et al., 2016c). Natural metabolites are good alternatives to synthetic drugs because synthetic drugs are effective but often cause side effects (Newman et al., 2003; Atanasov et al., 2015). Natural products from food and traditional medicine are safer to use because they do not cause side effects and can still improve health (Veeresham, 2012; Ekor, 2014).

To address these challenges, this review investigates the role of nutrients and natural products in aging. Researchers are studying natural metabolite functions that protect and repair aging cells at the molecular level (Choi and Friso, 2010; Yuan et al., 2016). These metabolites work to reduce oxidative stress, improve metabolism, and regulate aging-related genes (Harman, 1992; Chen et al., 2016). Recently aging studies aim to delay age-related conditions and reverse some cellular changes (López-Otín et al., 2013; Ristow and Schmeisser, 2014).

In this context, the paper focuses on how vitamins, minerals, antioxidants, and botanical drugs can prevent or reverse cellular damage caused by aging (Calder, 2017). These approaches represent a shift in healthcare, where the focus is moving from treating symptoms to preventing diseases (Kaeberlein et al., 2015). This review examines the increasing interest in using nutrients and natural products as potential drugs (Cragg and Newman, 2013; Atanasov et al., 2015). It also explains their important role in healthcare by analyzing recent research trends (Li et al., 2016c; Yuan et al., 2016).

This review focuses on vitamins and minerals with well-documented roles in aging-related pathways, particularly their effects on oxidative stress regulation, immune modulation, mitochondrial function, and neuroprotection. Metabolites were selected based on their mechanistic insights and clinical relevance, with priority given to randomized controlled trials (RCTs), preclinical studies, and systematic reviews/meta-analyses that provided mechanistic or clinical insights into aging interventions. Case reports, narrative reviews, and studies with insufficient methodological details were excluded.

Moreover, this review explores how natural products can be integrated into mainstream medical practices, while also addressing key challenges such as standardization, clinical validation, and regulatory approval. By adopting this approach, we aimed to provide a balanced evaluation of their therapeutic potential in aging-related pathways while discussing their translational challenges.

## 2 The role of nutrients in anti-aging drug development

Nutrients, the building blocks of life, play a crucial role in maintaining cellular health and preventing age-related decline (Kennedy et al., 2014; Ames, 2018). This section explores various vitamins, minerals, and antioxidants that have shown promise in slowing aging processes. Key nutrients like Vitamin C, Vitamin E, selenium, and omega-3 fatty acids are highlighted for their antioxidative and anti-inflammatory properties, which contribute to cellular longevity and resilience (Rayman, 2012; Li et al., 2016c; Calder, 2017). Recent studies illustrate how these nutrients not only prevent cellular damage but also enhance the efficacy of other therapeutic products, offering a dual advantage in anti-aging therapy (Traber and Atkinson, 2007; Ames, 2018). The discussion extends to the mechanisms through which these nutrients influence genetic pathways associated with aging, such as DNA repair and metabolic regulation (Harman, 2003; López-Otín et al., 2013). These nutrients and their effects and mechanisms are summarized in Table 1.

**TABLE 1:** Role of nutrients.

Nutrient	Effect	Mechanism
Omega-3 Fatty Acids	Cardiovascular Health	Improves endothelial function, reduces LDL oxidation
	Cognitive Health	Activates PI3K/Akt signaling, increases BDNF levels
	Anti-Inflammation	Produces resolvins, inhibits NF-κB
Vitamin A	Vision Support	Forms rhodopsin, maintains retinal health
	Immune Function	Strengthens epithelial barriers, enhances T-cell activity
	Cellular Regulation	Activates RARs/RXRs, regulates cell differentiation
	Anti-Inflammation	Modulates NF-κB, reduces chronic inflammation
Vitamin C	Antioxidant	Scavenges ROS, regenerates Vitamin E
	Immune Support	Enhances T-cell activity, boosts interferon production
	Cardiovascular Health	Activates eNOS, prevents LDL oxidation
Vitamin D	Bone Health	Enhances calcium absorption, strengthens bones
	Immune Regulation	Produces antimicrobial peptides, reduces inflammation
	Cognitive Support	Promotes neuronal repair, reduces neurodegeneration risk
Vitamin E	Antioxidant	Neutralizes ROS, prevents lipid peroxidation
	Cardiovascular Benefits	Improves endothelial function, reduces LDL oxidation
	Immune Support	Enhances T-cell function, reduces inflammatory cytokines
Vitamin K2	Bone Mineralization	Activates osteocalcin, promotes calcium binding in bone matrix
	Cardiovascular Health	Activates MGP to prevent arterial calcification, improves arterial elasticity
	Calcium Homeostasis	Regulates calcium distribution between bones and arteries
Coenzyme Q10	Energy Production	Facilitates ATP synthesis by supporting electron transport
	Antioxidant	Neutralizes ROS, protects mitochondrial membranes
	Cardiovascular Health	Enhances nitric oxide production, reduces lipid peroxidation
	Neuroprotection	Stabilizes mitochondria, reduces oxidative neuronal damage
Selenium	Antioxidant	Activates GPx, reduces ROS and oxidative stress
	Immune Support	Enhances T-cell proliferation, balances cytokine levels
	Cognitive Protection	Shields neurons, mitigates neuroinflammation
	Mitochondrial Health	Promotes fusion, prevents protein misfolding
Zinc	Immune Support	Enhances T-cell activity, balances cytokine production
	DNA Repair	Acts as a cofactor for DNA synthesis and repair enzymes
	Antioxidant	Stabilizes cell membranes, supports SOD activity
	Tissue Integrity	Regulates MMPs, prevents tissue stiffness and fibrosis

### 2.1 Omega-3 fatty acids

Omega-3 fatty acids are essential polyunsaturated fats primarily found in fish oil, flaxseeds, and walnuts (Calder, 2013). These fatty acids are known for their ability to reduce inflammation, support cardiovascular health, and enhance brain function, making them critical for healthy aging (Swanson et al., 2012; Calder, 2017). Their benefits arise mainly from their active forms, eicosapentaenoic acid (EPA) and docosahexaenoic acid (DHA) (Das, 2006). EPA serves as a precursor for anti-inflammatory eicosanoids, which inhibit pro-inflammatory mediators such as prostaglandins and leukotrienes (Serhan and Levy, 2018). DHA, as a structural metabolite of neuronal membranes, enhances membrane fluidity and modulates synaptic signaling, contributing to cognitive health and neuroprotection (Bazan, 2006). In the cardiovascular system, omega-3 fatty acids improve endothelial function by enhancing eNOS activity, which increases nitric oxide production and promotes vasodilation (Zgheel et al., 2014). They lower blood triglycerides, reduce low-density lipoprotein (LDL) oxidation, and suppress NF-κB signaling, contributing to cardiovascular health (Mozaffarian and Wu, 2011; Calder, 2017). In the brain, omega-3 fatty acids mitigate oxidative stress and neuroinflammation by reducing reactive oxygen species (ROS) and suppressing pro-inflammatory cytokines like IL-6 and TNF-α (Bazan, 2007). DHA activates PI3K/Akt signaling, supporting neuronal survival and reducing the risk of neurodegenerative conditions such as Alzheimer’s disease (Akbar et al., 2005; Bazan, 2006). Furthermore, omega-3 fatty acids influence brain-derived neurotrophic factor (BDNF) levels, promoting neurogenesis and cognitive (Cardoso et al., 2014; Dyall, 2015). These combined properties highlight their versatile role in promoting overall health and longevity (Simopoulos, 2008).

### 2.2 Vitamin A

Vitamin A (Retinoids), a fat-soluble vitamin found in foods like liver, carrots, and leafy green vegetables, plays a crucial role in vision, immune function, and cellular regulation (Blomhoff, 1994; Tanumihardjo, 2011). In vision, vitamin A is indispensable for forming rhodopsin, a light-sensitive pigment in the retina essential for low-light and color vision (Saari, 2012). It also maintains the health of the corneal epithelium and conjunctiva, reducing the risk of conditions like xerophthalmia and age-related macular degeneration (AMD) (Sommer, 2008). Retinoic acid, an active metabolite of vitamin A, regulates genes involved in photoreceptor differentiation and repair, preserving visual function with age (Saari, 2012). Vitamin A supports the immune system by strengthening epithelial barriers in the skin and mucous membranes, which act as the first line of defense against pathogens (Ross, 2012). It enhances the activity of T-cells and B-cells while promoting cytokine production essential for adaptive immunity (Stephensen, 2001; Ross, 2012). This is particularly vital in older populations, where weakened immunity heightens susceptibility to infections (Michaud et al., 2013). Additionally, vitamin A’s anti-inflammatory properties modulate NF-κB signaling, helping to reduce chronic inflammation associated with aging (Mora et al., 2008). At the cellular level, retinoic acid governs cell proliferation, differentiation, and apoptosis by activating nuclear receptors such as RARs (Retinoic Acid Receptors) and RXRs (Retinoid X Receptors) (Altucci and Gronemeyer, 2001). Retinoic acid also influences Wnt/β-catenin signaling, playing a key role in tissue regeneration and repair (Blum and Begemann, 2012).

### 2.3 Vitamin C

Vitamin C (Ascorbic acid), also known as ascorbic acid, is a water-soluble vitamin found in fruits and vegetables such as citrus fruits, strawberries, and bell peppers (Levine et al., 1996; Carr A. C. and Frei B., 1999). It plays a critical role in healthy aging through its antioxidant properties, immune support, and cardiovascular benefits (Carr and Maggini, 2017). As a potent antioxidant, vitamin C scavenges ROS and regenerates other antioxidants like vitamin E, preventing lipid peroxidation and DNA damage, which are key contributors to aging (Frei et al., 1989; Carr A. and Frei B., 1999). It also modulates inflammatory pathways by inhibiting NF-κB activation, reducing chronic inflammation in aging populations (Monacelli et al., 2017). In immune function, vitamin C enhances T-cell and macrophage activity, promoting pathogen clearance and adaptive immunity (Carr and Maggini, 2017). Additionally, it boosts interferon production, improving antiviral responses and reducing the severity of infections, particularly in older adults (Hemilä, 2017). Vitamin C supports cardiovascular health by activating eNOS to increase nitric oxide (NO) production, improving blood vessel dilation and reducing arterial stiffness (May and Harrison, 2013). Vitamin C prevents LDL cholesterol oxidation, reducing the risk of atherosclerosis (Frei, 1991). Additionally, clinical studies have shown its blood pressure-lowering effects, contributing to cardiovascular health (Juraschek et al., 2012).

### 2.4 Vitamin D

Vitamin D (Secosteroids), a fat-soluble vitamin, is synthesized in the skin through sunlight exposure and obtained from fatty fish, fortified foods, and supplements (Holick, 2007). It is crucial for bone health, immune regulation, and overall wellbeing, particularly in aging populations (Pludowski et al., 2013). In the immune system, vitamin D enhances innate immunity by stimulating antimicrobial peptides like cathelicidins to combat infections (Wang et al., 2004; Gombart et al., 2005). It modulates adaptive immunity by reducing excessive inflammation via NF-κB signaling, which is vital for preventing respiratory infections in older adults (Wang et al., 2004; Zhang et al., 2014). Vitamin D supports cardiovascular health by improving endothelial function and regulating the renin-angiotensin-aldosterone system (RAAS), lowering blood pressure and reducing arterial stiffness (Carrara et al., 2016; Al-Ishaq et al., 2021). Its anti-inflammatory effects further protect against vascular inflammation, a key factor in cardiovascular diseases (Norman and Powell, 2014; Yin and Agrawal, 2014). In the brain, vitamin D promotes neuronal survival and repair through neurotrophic factors (Khairy and Attia, 2021). Deficiency is linked to increased risks of neurodegenerative diseases and cognitive decline (Berridge, 2017; Feart et al., 2017). Supplementation may improve memory and in deficient individuals (Gowda et al., 2015; Pettersen, 2017).

### 2.5 Vitamin E

Vitamin E (Tocopherols) is a fat-soluble antioxidant primarily found in nuts, seeds, and vegetable oils (Bramley et al., 2000). It plays a crucial role in protecting cellular metabolites from oxidative damage and supports immune function and cardiovascular health, particularly during aging (Gombart et al., 2005; Traber and Stevens, 2011). Its primary function is to neutralize ROS and prevent lipid peroxidation, thereby safeguarding cell membranes from oxidative damage—a key contributor to aging and chronic diseases (Traber and Atkinson, 2007). Vitamin E also regenerates other antioxidants, such as vitamin C, to their active forms (Niki, 2014). In the immune system, Vitamin E enhances T-cell and natural killer (NK) cell function by stabilizing membranes and improving pathogen responsiveness. It reduces pro-inflammatory cytokines by inhibiting NF-κB signaling, mitigating chronic inflammation commonly seen in aging (Wu and Meydani, 2008). Vitamin E supports cardiovascular health by preventing LDL cholesterol oxidation, improving endothelial function, and reducing platelet aggregation (Freedman and Keaney, 2001; Meydani, 2001). These actions lower the risk of atherosclerosis, heart attacks, and strokes, particularly in individuals with low baseline levels of Vitamin E (Rimm et al., 1993). It also interacts with membrane lipids to optimize fluidity, promoting effective signal transduction for cellular communication (Traber and Packer, 1995; Zingg, 2019).

### 2.6 Vitamin K2

Vitamin K2 (Menaquinones) is a fat-soluble vitamin found in fermented foods, egg yolks, and dairy products (Khalil et al., 2021). It regulates calcium distribution to support bone and cardiovascular health, especially in aging populations (Hariri et al., 2021; Khalil et al., 2021). It activates osteocalcin, a protein responsible for binding calcium to the bone matrix, thereby improving bone mineral density and reducing the risk of fractures and osteoporosis (Na et al., 2022; Aaseth et al., 2024). Vitamin K2 also synergizes with vitamin D to support bone integrity and mitigate bone loss associated with aging (Kidd, 2010; Capozzi et al., 2020; Singh et al., 2022). In the cardiovascular system, vitamin K2 activates Matrix Gla Protein (MGP), which inhibits arterial calcium deposition (Shioi et al., 2020; Hariri et al., 2021). Vitamin K2 reduces vascular calcification and decreases the risk of atherosclerosis and cardiovascular diseases (Kurnatowska et al., 2015; Shioi et al., 2020; Hariri et al., 2021).

### 2.7 Coenzyme Q10 (CoQ10)

Coenzyme Q10 (CoQ10, Ubiquinone-10) is a lipid-soluble metabolite found in mitochondria, essential for cellular energy production and antioxidant defense (Turunen et al., 2004; Hidalgo-Gutiérrez et al., 2021). It is abundant in energy-demanding tissues like the heart, brain, and muscles but decreases with age, making it significant in aging research (López-Lluch, 2019; Gutierrez-Mariscal et al., 2021). CoQ10 supports ATP production by facilitating electron transfer in the mitochondrial electron transport chain (ETC) (Mantle and Dybring, 2020; Elgar, 2021). This ensures sufficient energy supply for tissues vulnerable to mitochondrial dysfunction during aging (Barcelos and Haas, 2019; Fišar and Hroudová, 2024). As a potent antioxidant, CoQ10 neutralizes ROS and protects mitochondrial membranes, proteins, and DNA from oxidative damage (Littarru and Tiano, 2007; Akbari et al., 2020). It also regenerates other antioxidants like vitamin E, enhancing overall antioxidant defense (Kagan et al., 2000). In the cardiovascular system, CoQ10 improves endothelial function by boosting nitric oxide (NO) production, promoting vasodilation, and reducing arterial stiffness (Rabanal-Ruiz et al., 2021). It inhibits lipid peroxidation, lowering the risk of atherosclerosis, and has been shown to improve heart function in heart failure patients (Rabanal-Ruiz et al., 2021). CoQ10 contributes to neuroprotection by reducing oxidative damage and improving mitochondrial efficiency in neurons (Young et al., 2007; Pradhan et al., 2021). These effects help maintain synaptic integrity and reduce the risk of neurodegenerative diseases such as Alzheimer’s and Parkinson’s (Kadian et al., 2022; Bagheri et al., 2023).

### 2.8 Selenium

Selenium is an essential trace element found in foods like Brazil nuts, seafood, and whole grains (Rayman, 2008). It acts as a cofactor for antioxidant enzymes, including glutathione peroxidase (GPx), and plays a crucial role in protecting cells from oxidative stress while supporting immune function (Beck et al., 2003; Santi et al., 2013). In the antioxidant system, selenium-dependent enzymes such as GPx reduce hydrogen peroxide and lipid hydroperoxides, preventing oxidative damage to lipids, proteins, and DNA (Ingold and Conrad, 2018; Weaver and Skouta, 2022). This action helps maintain cellular integrity and lowers the risk of age-related diseases, including cardiovascular and neurodegenerative disorders (Cai et al., 2019; Bjørklund et al., 2022a). Selenium also enhances the proliferation and function of T-cells and natural killer (NK) cells, modulating cytokine production to balance pro-inflammatory and anti-inflammatory responses (Hoffmann and Berry, 2008; Avery and Hoffmann, 2018). Emerging research suggests that selenium contributes to cognitive health by protecting neurons from oxidative damage and reducing neuroinflammation (Bai et al., 2024). Selenium-dependent enzymes like GPx and thioredoxin reductase help mitigate inflammation linked to neurodegenerative diseases such as Alzheimer’s and Parkinson’s (Bjørklund et al., 2022c; Oliveira et al., 2023). Beyond its antioxidant role, selenium influences mitochondrial dynamics by promoting mitochondrial fusion and preventing excessive fission, maintaining cellular energy balance (Kumari et al., 2012). Additionally, selenium-dependent enzymes help prevent protein misfolding, a key factor in neurodegenerative diseases (Cardoso et al., 2015; Zhang and Song, 2021).

### 2.9 Zinc

Zinc is an essential trace mineral found in foods such as meat, fish, and legumes (Fairweather-Tait, 1988). It is involved in various cellular processes, including enzymatic activity, DNA repair, and immune regulation, making it vital for healthy aging (Haase and Rink, 2009; Costa et al., 2023). In the immune system, zinc enhances the function of T-cells and natural killer (NK) cells, which are essential for adaptive and innate immunity (Shankar and Prasad, 1998). It also modulates cytokine production and prevents excessive inflammatory responses by inhibiting NF-κB signaling (Jarosz et al., 2017). This helps maintain immune balance and reduces chronic inflammation, a key contributor to aging-related diseases (Vasto et al., 2006; Wong et al., 2021). Zinc plays a crucial role in DNA repair and genomic stability by serving as a cofactor for enzymes such as DNA polymerase (Wu and Wu, 2023). By supporting DNA synthesis and repair, zinc helps prevent genomic instability, a key factor in cellular aging and age-related diseases (Dreosti, 2001; Costa et al., 2023). Zinc-finger transcription factors contribute to chromatin remodeling and gene regulation, further supporting cellular health during aging (Powell et al., 2019; Kamaliyan and Clarke, 2024). As an antioxidant, zinc stabilizes cell membranes and neutralizes ROS, preventing oxidative damage (Marreiro et al., 2017; Lee, 2018). It also supports the function of antioxidant enzymes like superoxide dismutase (SOD), which protects cells from oxidative stress (Mondola et al., 2016). This antioxidant role helps delay aging and lowers the risk of neurodegenerative and cardiovascular diseases (Prasad, 2014). Zinc regulates cellular senescence by modulating matrix metalloproteinases (MMPs), which remodel extracellular matrix metabolites and prevent tissue stiffness and fibrosis, common in aging tissues (Cabral-Pacheco et al., 2020).

## 3 Natural products as innovators in aging therapeutics

Natural products, including botanical drugs and phytochemicals, have been used for centuries in traditional medicine, but now gaining prominence in modern pharmacology for their anti-aging properties (Bjørklund et al., 2022b). This paragraph examines key natural products such as resveratrol, curcumin, and ginkgo biloba, focusing on their roles in modulating age-related pathways (Pyo et al., 2020; Assi et al., 2023). Research findings are discussed that demonstrate how these products not only mitigate oxidative stress and inflammation but also activate longevity genes such as sirtuins and AMP-activated protein kinase (AMPK) (Shehzad et al., 2011; Chung et al., 2012; Li X. et al., 2020). This review also explores the potential of these natural products to reverse or delay aging symptoms. These natural products and their effects and mechanisms are summarized in Table 2.

**TABLE 2:** Role of natural products.

Natural products	Effect	Mechanism
Resveratrol	Longevity Support	Activates SIRT1, enhances mitochondrial function
	Cardiovascular Health	Improves endothelial function, reduces LDL oxidation
	Neuroprotection	Reduces oxidative stress, protects neurons from inflammation
	Anti-Inflammatory Action	Inhibits NF-κB, lowers pro-inflammatory cytokines
Curcumin	Neuroprotection	Promotes amyloid clearance, reduces oxidative stress
	Anti-Inflammation	Inhibits NF-κB and COX-2 pathways, lowers cytokine levels
	Antioxidant	Scavenges ROS, boosts antioxidant enzyme activity
	Mitochondrial Health	Induces mitophagy through PINK1/Parkin pathway
Quercetin	Antioxidant	Scavenges ROS, prevents oxidative damage to lipids and DNA.
	Anti-Inflammation	Inhibits NF-κB, reduces pro-inflammatory cytokines
	Cardiovascular Health	Enhances endothelial function, reduces LDL oxidation
	Neuroprotection	Protects neurons, reduces oxidative stress and inflammation
Ginseng	Stress Reduction	Modulates HPA axis, lowers cortisol levels
	Cognitive Enhancement	Protects neurons, improves synaptic plasticity
	Antioxidant	Reduces ROS, enhances cellular resilience
	Immune Support	Enhances NK cell activity, strengthens immune defenses
Kaempferol	Antioxidant	Scavenges ROS, protects cells from oxidative damage
	Anti-Inflammation	Inhibits NF-κB and MAPK pathways, reduces cytokine levels
	Neuroprotection	Enhances BDNF expression, supports synaptic plasticity
Apigenin	Antioxidant	Neutralizes ROS, prevents lipid and DNA oxidation
	Anti-Inflammation	Inhibits NF-κB, reduces pro-inflammatory cytokines
	Neuroprotection	Enhances BDNF expression, supports neuronal survival
Green Tea Extract	Antioxidant	Neutralizes ROS, protects DNA and lipids from oxidative damage
	Anti-Inflammation	Inhibits NF-κB, reduces pro-inflammatory cytokines
	Cardiovascular Health	Enhances endothelial function, reduces LDL oxidation
	Neuroprotection	Protects neurons, reduces oxidative stress and neuroinflammation
Alpha-Lipoic Acid	Metabolic Regulations	Activates AMPK, enhances glucose uptake and insulin sensitivity
	Antioxidant	Scavenges free radicals, regenerates antioxidants
	Anti-Inflammation	Modulates NF-κB signalin, reduces inflammation, protects tissues
Astaxanthin	Antioxidant	Neutralizes ROS, protects lipids, proteins, and DNA.
	Neuroprotection	Reduces neuroinflammation, supports neuronal survival
	Cardiovascular Health	Prevents LDL oxidation, enhances endothelial function
	Skin Health	Reduces UV-induced damage, improves skin elasticity

### 3.1 Resveratrol

Resveratrol (3,5′,4′-trihydroxystilbene, [Polygonaceae]) is a polyphenolic metabolite found in red wine, grapes, and berries (Bonnefont-Rousselot, 2016). It is widely recognized for its antioxidant and anti-inflammatory properties and has been extensively studied for its role in promoting longevity and preventing age-related diseases (Bonnefont-Rousselot, 2016; Pyo et al., 2020). Resveratrol exerts its effects by scavenging ROS, thereby reducing oxidative stress and protecting lipids, proteins, and DNA from oxidative damage (Truong et al., 2018; Koushki et al., 2020). Since oxidative stress is a key contributor to aging and chronic diseases such as cardiovascular and neurodegenerative disorders, resveratrol’s antioxidant function plays a critical role in maintaining cellular health (Meng et al., 2020; Leyane et al., 2022). At the molecular level, resveratrol activates SIRT1 (Sirtuin 1), a key regulator of cellular metabolism, DNA repair, and cell survival (Borra et al., 2005; Jeong et al., 2007). By stimulating SIRT1, resveratrol mimics the effects of calorie restriction, enhancing mitochondrial function and promoting longevity (Rogina and Tissenbaum, 2024). Additionally, resveratrol increases mitochondrial biogenesis through the PGC-1α pathway, improving energy efficiency and reducing oxidative damage in aging cells (Higashida et al., 2013; Zhou et al., 2021). It also modulates the NAD+/NADH ratio, an essential factor in cellular metabolism, promoting adaptive stress responses and delaying cellular senescence (Jang et al., 2012; Desquiret-Dumas et al., 2013). Beyond its role in longevity, resveratrol has significant cardiovascular benefits. It improves endothelial function, enhances nitric oxide (NO) production, and increases blood flow, reducing the risk of heart disease (Xia et al., 2014). Additionally, it lowers LDL cholesterol oxidation, a critical factor in atherosclerosis, and improves blood vessel elasticity (Frémont et al., 1999; Cherniack and Troen, 2013; Breuss et al., 2019). Moreover, resveratrol exhibits anti-inflammatory properties by inhibiting NF-κB signaling, a central mediator of chronic inflammation (Ma et al., 2015; Meng et al., 2021).

### 3.2 Curcumin

Curcumin (*Curcuma longa* L., [Zingiberaceae]), the active metabolite in turmeric (Curcuma longa), is well known for its anti-inflammatory, antioxidant, and neuroprotective properties (Silvestro et al., 2021; Genchi et al., 2024). It has been extensively studied for its potential therapeutic effects in aging and age-related diseases (Sikora et al., 2010; Nunes et al., 2024). Curcumin exerts its anti-inflammatory effects primarily by inhibiting key inflammatory pathways such as NF-κB and COX-2, which regulate the production of pro-inflammatory cytokines and mediators involved in chronic inflammation (Yuan et al., 2018; Fu et al., 2022). By reducing inflammation, curcumin helps lower the risk of age-related conditions, including arthritis and cardiovascular diseases (He et al., 2015). As an antioxidant, curcumin neutralizes ROS and enhances cellular defense mechanisms (Sathyabhama et al., 2022). It stimulates antioxidant enzymes such as superoxide dismutase (SOD) and glutathione peroxidase, which protect cells from oxidative stress, a key driver of aging and neurodegenerative diseases (Khayatan et al., 2024). In the brain, curcumin’s antioxidant and anti-inflammatory properties contribute to its neuroprotective effects (Genchi et al., 2024). It mitigates oxidative damage and neuroinflammation, both implicated in Alzheimer’s disease, and promotes the clearance of amyloid plaques by activating autophagy and modulating microglial activity (Azzini et al., 2024). Curcumin also plays a crucial role in mitochondrial quality control by inducing mitophagy through the PINK1/Parkin pathway, thereby preventing age-related cellular decline (Cao et al., 2020; Jin et al., 2022). Additionally, it activates the Nrf2-Keap1 pathway, enhancing the production of endogenous antioxidant enzymes to counteract oxidative stress (Soetikno et al., 2013; Lin et al., 2019).

### 3.3 Querectin

Quercetin (3,3′,4′,5,7-Pentahydroxyflavone, [Fagaceae/Rutaceae]) is a flavonoid found in apples, onions, and green tea. It has strong antioxidant and anti-inflammatory properties, making it useful for aging and age-related diseases (Li et al., 2016b; Deepika and Maurya, 2022). Quercetin neutralizes ROS and reduces oxidative stress in cells, protecting them from damage (Costa et al., 2016). In the immune system, quercetin reduces inflammation by inhibiting the NF-κB pathway, which regulates pro-inflammatory cytokine production (Comalada et al., 2005). This makes quercetin useful in controlling chronic inflammation, especially in aging populations (David et al., 2016). Quercetin improves vascular health by enhancing blood vessel function and reducing LDL cholesterol oxidation, which helps prevent atherosclerosis (Jiang et al., 2020). It also improves circulation and lowers blood pressure (Serban et al., 2016). Studies show that quercetin may protect the brain from oxidative damage and neuroinflammation, both of which contribute to neurodegenerative diseases like Alzheimer’s (Khan et al., 2019). By crossing the blood-brain barrier, it helps sustain brain health throughout aging (Grewal et al., 2021). Quercetin also exerts its anti-aging effects by inhibiting the PI3K/AKT/mTOR pathway, which regulates cellular growth and aging (Granato et al., 2017; Li et al., 2018). By modulating this pathway, quercetin promotes autophagy, prevents cellular senescence, and strengthens intercellular junctions to enhance tissue integrity while reducing inflammation-induced damage (Rather and Bhagat, 2020).

### 3.4 Ginseng

Ginseng (*panax ginseng*, C.A.Mey., [Araliaceae]) is a well-known botanical drug used in traditional medicine for its adaptogenic properties, modulating immune functions and enhancing resilience to various stressors (Ratan et al., 2021). The active metabolites in ginseng, particularly ginsenosides, are responsible for its medicinal effects (Leung and Wong, 2010). Ginseng modulates the hypothalamic-pituitary-adrenal (HPA) axis, which regulates the body’s stress response (Lee and Rhee, 2017). By reducing the secretion of cortisol, a stress hormone, ginseng helps lower chronic inflammation and balance hormonal levels (Liao et al., 2018). In the immune system, ginseng enhances the activity of macrophages, T-cells, and natural killer (NK) cells, all of which are crucial for fighting infections and maintaining immune homeostasis (He et al., 2017). Ginsenosides activate pathways such as NF-κB and MAPK, which are involved in immune response and inflammation regulation, helping to strengthen the body’s defense mechanisms (Kim et al., 2017). Ginseng also supports cognitive function by protecting neurons from oxidative stress and improving synaptic plasticity (Shin et al., 2024). Ginseng’s active metabolites, ginsenosides, modulate AMPK signaling to enhance cellular energy sensing and lipid metabolism (Wang et al., 2022). This mechanism not only combats age-related metabolic disorders but also reduces chronic low-grade inflammation (Fan et al., 2020). Ginseng has also been shown to promote angiogenesis through VEGF signaling, aiding tissue repair and regeneration (Song et al., 2023).

### 3.5 Kaempferol

Kaempferol (3,4′,5,7-Tetrahydroxyflavone [Fabaceae/Brassicaceae]) is a flavonoid found in a variety of fruits, vegetables, and beverages, including broccoli, kale, beans, and tomato (Calderon-Montano et al., 2011). It has been widely studied for its antioxidant, anti-inflammatory, and anti-cancer properties, making it a promising metabolite for promoting health, particularly in aging (Gutiérrez-del-Río et al., 2016; Shahbaz et al., 2023). Kaempferol acts as a potent antioxidant by neutralizing ROS and preventing oxidative damage to cells (Wu et al., 2018). By scavenging free radicals, kaempferol reduces oxidative stress, which is a major contributor to aging and the development of chronic diseases, such as cardiovascular disease, cancer, and neurodegenerative disorders (Liao et al., 2016; Rahul and Siddique, 2021; Kamisah et al., 2023). In terms of inflammation, kaempferol modulates inflammatory pathways, particularly by inhibiting NF-κB and MAPK signaling, which are involved in the production of pro-inflammatory cytokines (Wang et al., 2020). In neuroprotection, kaempferol has been shown to protect neurons from oxidative stress and reduce neuroinflammation (Nezhad Salari et al., 2024). It may help in maintaining cognitive function by promoting the production of BDNF, which is involved in neuronal survival and synaptic plasticity (Silva dos Santos et al., 2021). Kaempferol inhibits the senescence-associated secretory phenotype (SASP) by reducing the production of inflammatory cytokines such as IL-6 and TNF-α (Lim et al., 2015; Hussain et al., 2024).

### 3.6 Apigenin

Apigenin (4′,5,7-Trihydroxyflavone) [Asteraceae] is a flavonoid found in a variety of plants, including parsley, celery, chamomile, and basil (Salehi et al., 2019b). Known for its antioxidant, anti-inflammatory, and anti-cancer properties, apigenin has been studied for its potential health benefits, particularly in aging (Madunić et al., 2018). Apigenin acts as a potent antioxidant by scavenging ROS and reducing oxidative stress in cells (Jung, 2014). In terms of inflammation, apigenin inhibits the NF-κB pathway, which is involved in the production of pro-inflammatory cytokines such as IL-1β, IL-6, and TNF-α (Patil et al., 2016). By modulating this pathway, apigenin helps reduce chronic inflammation, a common contributor to diseases like arthritis, heart disease, and neurodegenerative disorders in aging populations (Ali et al., 2017). In neuroprotection, apigenin has been shown to reduce oxidative stress and inflammation in the brain (Li R. et al., 2016). It increases the expression of BDNF, which is important for neuronal survival and synaptic plasticity (Sharma et al., 2020; Gao et al., 2023). These effects help maintain cognitive function and may reduce the risk of neurodegenerative diseases like Alzheimer’s (Zhao et al., 2013). Apigenin enhances mitochondrial function through increased expression of SOD2, reducing mitochondrial-derived oxidative stress (Jung, 2014; Pal et al., 2017). Apigenin’s ability to stabilize transcription factors like FOXO3 further contributes to its longevity-enhancing effects (Kawasaki et al., 2010; Lin et al., 2015).

### 3.7 Green tea extract

Green Tea Extract (*Camellia sinensis* (L.) Kuntze) [Theaceae] is derived from the leaves of Camellia sinensis and is rich in polyphenols, particularly epigallocatechin gallate (EGCG), which is the main natural products (Komes et al., 2010; Du et al., 2012). It is well-known for its antioxidant, anti-inflammatory, and metabolism-enhancing properties, making it a popular natural product for promoting healthy aging (Chen et al., 2009; Senanayake, 2013; Ohishi et al., 2016). Green tea extract exhibits strong antioxidant activity by neutralizing ROS and enhancing the activity of endogenous antioxidant enzymes (Elbling et al., 2005). The extract also exerts anti-inflammatory effects by inhibiting pathways like NF-κB and reducing levels of pro-inflammatory cytokines such as IL-6 and TNF-α (Ohishi et al., 2016). In addition, EGCG from green tea extract improves mitochondrial function and enhances energy metabolism by activating pathways like AMPK, which promotes cellular energy balance (Pournourmohammadi et al., 2017; Ha et al., 2018). Green tea extract also supports cognitive health by protecting neurons from oxidative stress and reducing neuroinflammation (Prasanth et al., 2019; Valverde-Salazar et al., 2023). Studies have shown its ability to enhance memory and learning in aging populations (Mancini et al., 2017). Green tea extract, rich in EGCG, enhances the DNA damage response (DDR) by upregulating ATM and ATR kinases, which repair double-strand breaks (Kuo et al., 2016; Majidinia et al., 2019). Additionally, EGCG modulates circadian clock genes, improving metabolic and cognitive functions in aging individuals (Liu et al., 2023).

### 3.8 Alpha-Lipoic Acid (ALA)

Alpha-Lipoic Acid (1,2-Dithiolane-3-pentanoic acid) (ALA) is a natural metabolite with strong antioxidant properties, crucial for energy metabolism and cellular protection (Tibullo et al., 2017; Superti and Russo, 2024). It is found in small amounts in foods like vegetables, futis and red meat (Salehi et al., 2019a). ALA’s water- and fat-solubility allows it to act in various cellular compartments, where it scavenges free radicals and regenerates other antioxidants, contributing to healthy aging (Shay et al., 2009; Nobakht-Haghighi et al., 2018). Additionally, ALA modulates NF-κB signaling, reducing inflammation and protecting tissues from chronic damage (Chang et al., 2017; Ishii et al., 2017). In metabolic health, ALA enhances insulin sensitivity by activating AMPK (AMP-activated protein kinase), which improves glucose uptake in muscle cells and lowers blood sugar levels (Lee et al., 2005; Targonsky et al., 2006; Shen et al., 2007).

### 3.9 Astaxanthin

Astaxanthin (3,3′-Dihydroxy-β,β-carotene-4,4′-dione) [Haematococcaceae] is a carotenoid pigment primarily found in microalgae, salmon, and krill (Johnson and An, 1991). It is known for its powerful antioxidant and anti-inflammatory properties, which contribute to its benefits for aging, skin health, and overall wellbeing (Pereira et al., 2021; Chae et al., 2022). As a powerful antioxidant, astaxanthin is significantly stronger than other carotenoids such as β-carotene and lutein (Naguib, 2000; Yuan et al., 2011; Ambati et al., 2014). It neutralizes ROS and protects cells from oxidative damage by scavenging free radicals (Mohammadi et al., 2021). Its unique structure enables it to safeguard both the lipid and aqueous phases of cells, effectively protecting various cellular metabolites, including cell membranes, proteins, and DNA (Ambati et al., 2014; Brotosudarmo et al., 2020). In addition to its antioxidant effects, astaxanthin exhibits anti-inflammatory properties by inhibiting key signaling pathways, including NF-κB and COX-2 (Choi et al., 2008; Farruggia et al., 2018). These pathways are involved in the production of pro-inflammatory cytokines and mediators, which contribute to chronic inflammation (Lee et al., 2003). By modulating these pathways, astaxanthin helps prevent the progression of inflammation-related diseases such as arthritis, cardiovascular disease, and neurodegeneration (Chang and Xiong, 2020). Astaxanthin also supports eye health by reducing oxidative stress and inflammation in the retina (Yeh et al., 2016; Giannaccare et al., 2020). It protects retinal cells from damage caused by light exposure and helps prevent AMD, a leading cause of vision loss in older adults (Otsuka et al., 2013; Giannaccare et al., 2020; Alugoju et al., 2023). Additionally, astaxanthin improves blood flow to the eyes, further enhancing visual function (Giannaccare et al., 2020).

## 4 Upcoming trends and challenges

As research continues to uncover the vast potential of nutrients and natural products in promoting health and longevity, several emerging trends and challenges are shaping the future of anti-aging therapies (Ros and Carrascosa, 2020; Bjørklund et al., 2022b). These developments highlight the importance of a holistic and evidence-based approach to leveraging natural metabolites for sustainable health benefits.

### 4.1 Personalized nutrition and precision medicine

One of the most promising trends is the move towards personalized nutrition and precision medicine. Advances in genomics and metabolomics are enabling a deeper understanding of individual differences in nutrient metabolism and response (Kaput and Rodriguez, 2006; Hadi, 2023). This personalized approach allows for tailored dietary and supplementation strategies that align with a person’s genetic profile, lifestyle, and health status, optimizing the efficacy of natural anti-aging interventions (Biesalski et al., 2009; Afshin et al., 2019). The integration of artificial intelligence and big data analytics further enhances the ability to develop customized health plans that promote longevity and mitigate age-related conditions (Topol, 2019).

Several clinical applications of precision medicine in nutrition are already in use (Wishart, 2016). Nutrigenomics-based dietary interventions enable personalized dietary recommendations based on genetic variants affecting nutrient metabolism, such as MTHFR polymorphisms influencing folate metabolism (Kiani et al., 2022; Andrade et al., 2025). Metabolomics-guided supplementation tailors nutrient intake by analyzing an individual’s metabolic profile to identify micronutrient deficiencies and optimize supplementation strategies (Rigamonti et al., 2023). Additionally, biomarker-driven antioxidant therapy assesses oxidative stress markers, such as the glutathione (GSH/GSSG) ratio, to guide personalized antioxidant interventions aimed at reducing cellular damage and improving metabolic health (Yagishita et al., 2020; Pan et al., 2024). These advancements illustrate how precision medicine strategies are being integrated into clinical practice to enhance the effectiveness of nutrient-based interventions (Bailey and Stover, 2023; Keijer et al., 2024).

### 4.2 Synergistic formulations

Another trend is the development of synergistic formulations that combine multiple nutrients and natural products to enhance their collective benefits (Ebrahimi et al., 2023). Research is increasingly focusing on the synergistic effects of metabolites such as vitamins, polyphenols, and omega-3 fatty acids (Liu, 2003; Li et al., 2016c; Calder, 2017; Ebrahimi et al., 2023). These combinations can potentially offer more significant health benefits than individual metabolites by targeting multiple pathways involved in aging (Liu, 2003). Formulating these combinations into easily accessible supplements and functional foods can provide practical solutions for aging populations seeking to maintain their health (Cencic and Chingwaru, 2010).

### 4.3 Advances in delivery systems

Many natural metabolites suffer from poor bioavailability due to low solubility, rapid metabolism, and degradation, limiting their therapeutic efficacy. Addressing these challenges, improving the bioavailability and efficacy of natural metabolites through advanced delivery systems is a crucial area of innovation (Obeid et al., 2017).

*In vivo* bioavailability is often restricted by multiple factors, including poor water solubility, instability in the gastrointestinal (GI) tract, extensive first-pass metabolism, and rapid systemic clearance. For instance, curcumin, a well-known polyphenol, has poor absorption due to its hydrophobic nature and rapid hepatic metabolism (Nelson et al., 2017). Coenzyme Q10 (CoQ10) also exhibits poor bioavailability due to its high molecular weight and low water solubility, which limit its intestinal absorption and systemic circulation (Zhang et al., 2022). Polyphenols such as quercetin and resveratrol also face challenges related to instability and rapid conjugation, leading to reduced systemic bioavailability (El Monfalouti and Kartah, 2024).

To overcome these limitations, several advanced drug delivery technologies are being explored. Nanoencapsulation improves the solubility and stability of hydrophobic metabolites, facilitating better absorption (Livney, 2016). Liposomal delivery systems, composed of phospholipid bilayers, protect active metabolites from enzymatic degradation and enhance their permeability across biological membranes (Singh et al., 2019). Solid lipid nanoparticles and nanostructured lipid carriers further enhance drug stability and prolong systemic circulation, improving therapeutic efficacy (Safta et al., 2024).

Another promising approach is the use of cyclodextrin complexes, which form inclusion complexes with poorly soluble metabolites, increasing their water solubility and enhancing bioavailability (Loftsson and Brewster, 2012; Kurkov and Loftsson, 2013). Prodrug strategies, which involve modifying the chemical structure of a metabolite to improve its absorption and metabolic stability, are also gaining attention in the field of natural product-based therapeutics (Rautio et al., 2008).

These technologies can overcome the limitations of traditional supplementation, ensuring that active metabolites reach their target tissues at therapeutic levels. Further research is needed to refine these delivery strategies and optimize their application in clinical settings to maximize the therapeutic potential of natural metabolites.

### 4.4 Regulatory and quality assurance challenges

Despite the potential benefits, the widespread adoption of nutrient and natural product-based therapies faces significant regulatory and quality assurance challenges (Yadav et al., 2024). Ensuring the safety, efficacy, and consistency of these products requires stringent regulatory oversight and robust quality control measures (Bailey et al., 2013). The variability in the composition of natural products, influenced by factors such as sourcing, processing, and storage, necessitates standardized practices to guarantee their therapeutic potential (Kunle et al., 2012).

In addition, the interaction between natural metabolites and conventional medications remains a critical concern. Some nutrients and phytochemicals can influence drug metabolism by modulating cytochrome P450 enzymes, leading to altered drug efficacy or toxicity (Holst and Williamson, 2008; Johnson, 2008). For instance, St. John’s Wort induces CYP3A4, reducing the plasma concentration of certain drugs, whereas grapefruit juice inhibits the same enzyme, leading to potential drug accumulation and adverse effects (Dürr et al., 2000; Dahan and Altman, 2004; Rahimi and Abdollahi, 2012). Moreover, excessive intake of fat-soluble vitamins, iron, or polyphenols may lead to toxicity or interfere with drug absorption (Martin and Appel, 2009; Awuchi et al., 2020). Addressing these risks requires comprehensive safety assessments and clearer guidelines on the co-administration of natural products with pharmaceuticals (Colalto, 2010).

Furthermore, the lack of standardized labeling and dosage recommendations for natural supplements increases the risk of misuse (Avigan et al., 2016; Bailey, 2020). Unlike pharmaceuticals, where dosing is strictly regulated, natural metabolites often exhibit dose-dependent effects, making it challenging to establish universally safe and effective intake levels (Hasler, 2002; Wen et al., 2021). Certain natural products may also be contraindicated for individuals with specific health conditions. For example, high doses of omega-3 fatty acids may increase bleeding risk in individuals taking anticoagulants (Kapoor et al., 2021). Regulatory agencies must work towards harmonized guidelines that define safe consumption limits and potential contraindications, ensuring that natural product-based therapies are both effective and safe for consumers (Bast et al., 2002).

### 4.5 Ethical and accessibility considerations

As the popularity of anti-aging therapies grows, ethical and accessibility considerations must be addressed (Trothen, 2024). Ensuring that advancements in personalized nutrition and natural therapies are accessible to diverse populations, including underserved communities, is vital for promoting health equity (Petersen and Kwan, 2011). Additionally, ethical considerations regarding the use of genetic information and personalized health data must be carefully managed to protect individual privacy and autonomy (Vayena et al., 2018).

Furthermore, economic and environmental challenges must be considered when evaluating the scalability of natural metabolite-based interventions. Certain high-value natural metabolites, such as fish-derived omega-3 fatty acids and rare plant extracts, pose challenges in terms of cost, availability, and sustainability (Venegas-Calerón et al., 2010). The extraction and production of these metabolites often require significant resources, leading to high consumer prices and potential supply chain limitations (Palma et al., 2016). Additionally, overharvesting of rare botanical sources and intensive aquaculture practices for omega-3 production can contribute to ecological imbalances and biodiversity loss (Falkenberg et al., 2020; Zhang et al., 2024). To mitigate these challenges, research is increasingly focusing on alternative sources, such as microalgae-derived omega-3 and biotechnologically synthesized plant metabolites, which offer more sustainable and scalable solutions (Liu et al., 2022; Hassan Zadeh, 2024). Addressing these economic and environmental barriers is crucial for ensuring that natural therapies remain viable and accessible to a broader population.

### 4.6 Clinical trials investigating nutrients and natural products for age-related diseases

Several clinical trials have investigated the efficacy of nutrients and natural products in treating age-related diseases, including ocular, neurological, and muscular disorders (Dai et al., 2010; Suzuki et al., 2013; Dysken et al., 2014; Giannaccare et al., 2019; Voulgaropoulou et al., 2019). These studies provide critical insights into the therapeutic potential and limitations of natural metabolites in clinical settings. Table 3 summarizes key trials assessing the impact of these products on human health. While many studies demonstrate promising effects of natural products in *in vitro* models, these findings require further validation through well-designed *in vivo* and clinical studies to establish translational relevance (Denayer et al., 2014; Sorkin et al., 2020). While some studies demonstrate promising benefits, such as improved cognitive function and reduced oxidative stress, further large-scale, long-term trials are needed to confirm their effectiveness and establish optimal dosing strategies.

**TABLE 3:** Clinical trials on nutrients and natural products for age-related diseases.

Nutrient/Natural product	Disease	Key findings	Model	Dose	References
Omega-3 Fatty Acids	Dry Eye Disease	Improves dry eye symptoms, tear film stability, and tear production	Clinical (RCTs)	Eicosapentaenoic acid (EPA) 128∼1,440 mg Docosahexaenoic acid (DHA) 99∼1,050 mg	Brignole-Baudouin et al. (2011), Bhargava et al. (2013), Bhargava and Kumar (2015), Bhargava et al. (2016a), Bhargava et al. (2016b), Giannaccare et al. (2019)
Vitamin D	Alzheimer’s disease, Parkinson’s disease	Enhanced cognitive function, Stabilized Parkinson’s disease	Clinical (RCTs)	1200 IU/d, 2000 IU/d	Annweiler and Beauchet (2011), Annweiler et al. (2012), Suzuki et al. (2013)
Vitamin E	Alzheimer’s Disease	Slowed functional decline in Alzheimer’s disease	Clinical (RCTs)	1000 IU twice a day	Sano et al. (1997), Grundman (2000), Dysken et al. (2014)
Coenzyme Q10	Cardiovascular disease, Parkinson’s disease	Improved mitochondrial function and vascular health in heart failure Slowed functional decline in early Parkinson’s with 1,200 mg/day CoQ10	Clinical (RCTs)	200∼1,200 mg/day	Shults et al. (2002), Mortensen et al. (2014)
Curcumin	Alzheimer’s disease	Enhanced cognitive function	Clinical (RCTs)	300∼1,500 mg/day	Hishikawa et al. (2012), Cox et al. (2015), Rainey-Smith et al. (2016), Voulgaropoulou et al. (2019)

### 4.7 Essential amino acids in aging

Beyond vitamins, minerals, and natural products, essential amino acids also contribute to aging-related processes, particularly in muscle maintenance, metabolic regulation, and immune function (Roth, 2007). Branched-chain amino acids (BCAAs) such as leucine, isoleucine, and valine have been shown to support protein synthesis and mitochondrial function, while other amino acids like arginine and methionine play roles in oxidative stress modulation and immune response (Nie et al., 2018; Dai et al., 2020). While this review primarily focuses on non-protein nutrients, future research should explore the potential synergistic effects of essential amino acids with vitamins and natural metabolites in aging interventions.

### 4.8 Study limitations and research challenges

While numerous studies support the beneficial effects of nutrients and natural products in aging-related processes, many face limitations such as small sample sizes, lack of long-term follow-up, and variability in study designs, including differences in formulations, treatment duration, and administration routes (Ziegler et al., 2009; Puri et al., 2022; Li and Wang, 2024; Wimalawansa, 2025). Additionally, heterogeneity in study populations, inconsistent methodologies, and potential biases in self-reported dietary intake challenge the generalizability of findings (Behmer et al., 2002; Carocho and Ferreira, 2013; Mirmiran et al., 2021). Some clinical trials report positive outcomes, but confounding factors such as lifestyle, genetic variability, and concurrent interventions may influence results (Davey Smith and Ebrahim, 2003; Welch et al., 2011). Furthermore, preclinical studies, including In silico and *In vitro* models, provide valuable mechanistic insights but have inherent limitations in predicting clinical efficacy (Wu et al., 2020; Vashishat et al., 2024). Computational models rely on algorithm-based predictions that may not fully capture the complex biochemical interactions occurring in human physiology (Southern et al., 2008; Lee and Hu, 2019). Likewise, *In vitro* studies often utilize simplified cellular systems that lack systemic metabolic interactions, immune responses, and tissue-specific effects, making it difficult to extrapolate findings to whole-body physiology (Cook et al., 2012; McGonigle and Ruggeri, 2014).

A major controversy in the field is the lack of consensus on the effective doses of natural metabolites, which complicates their clinical translation (Singh et al., 2015). Unlike pharmaceuticals with well-defined dosing parameters, natural products often exhibit dose-dependent effects influenced by formulation, bioavailability, and individual metabolic differences (Boullata, 2005). For instance, curcumin requires high doses to achieve therapeutic effects due to its poor bioavailability, whereas lower doses may be sufficient for other polyphenols (Mahran et al., 2017; Abd El-Hack et al., 2021). Additionally, discrepancies in study methodologies, including variability in outcome measurements and data analysis approaches, contribute to inconsistent conclusions across studies (Kuhl, 2005; Argyropoulou et al., 2013; Sorkin et al., 2016). Many preclinical studies rely on *in vitro* models or animal experiments that do not fully replicate human physiological conditions, limiting their direct applicability (Henderson et al., 2013). While *In vitro* assays can provide initial screening for bioactivity, they lack the systemic complexity required to assess pharmacokinetics, organ-specific metabolism, and potential toxicity (Lipscomb and Poet, 2008; Astashkina et al., 2012). Therefore, findings from these models should be interpreted cautiously and validated through well-designed *In vivo* and clinical studies (Denayer et al., 2014).

In clinical trials, differences in intervention protocols, inconsistencies in sample selection, and inadequate control groups often result in conflicting findings (Jüni et al., 2001). Moreover, a lack of standardization in study endpoints and assessment methods further complicates comparative analysis, making it difficult to derive definitive conclusions about the efficacy of natural metabolites (Ziegelmann et al., 2020).

Another critical limitation is the insufficient understanding of the pharmacokinetics and bioavailability of many natural products (Hao et al., 2014; Pathak and Raghuvanshi, 2015). Variability in formulation types (e.g., whole extracts vs isolated metabolites), administration routes, and metabolic differences among individuals can significantly alter their biological activity (Possemiers et al., 2011; Sova and Saso, 2020). Without standardized dosing guidelines, it remains challenging to translate preclinical findings into effective therapeutic applications (Ioannidis et al., 2018).

Addressing these challenges requires standardized pharmacokinetic profiling and dose-response studies to establish optimal therapeutic windows for natural metabolites (Zeng et al., 2022). Future studies should also prioritize multicenter RCTs with larger and more diverse populations to improve the robustness of findings (Li W. et al., 2020). Furthermore, long-term clinical trials are necessary to determine the sustained effects and safety profiles of these metabolites over extended periods (Reginster et al., 2001; Zhou Y. et al., 2016). Given the variability in bioavailability and metabolic responses, future research should explore personalized approaches integrating metabolomics and nutrigenomics to tailor interventions to individual needs (Vyas et al., 2018; Lagoumintzis and Patrinos, 2023). Additionally, studies should focus on the synergistic effects of multiple natural metabolites, as combination therapies may enhance efficacy through complementary mechanisms (HemaIswarya and Doble, 2006; Zhou X. et al., 2016). Advanced drug delivery systems, such as nanoformulations and liposomal encapsulation, should also be investigated to improve bioavailability and therapeutic potential (Allen and Cullis, 2013; Khan et al., 2013). Additionally, *In silico*/*In vitro* methodologies should be supplemented with well-structured translational research strategies, ensuring that mechanistic findings can be effectively validated in physiologically relevant models (Bai et al., 2018; Rowland et al., 2018). Emerging technologies such as organ-on-a-chip systems and advanced 3D cell cultures may provide more predictive data, bridging the gap between early-stage research and clinical applications (Zhang and Radisic, 2017; Cavero et al., 2019). To establish more definitive conclusions, well-designed, large-scale, long-term clinical trials are needed (Piantadosi, 2024).

### 4.9 As the popularity future research directions

Ongoing research is critical to fully elucidate the mechanisms underlying the anti-aging effects of nutrients and natural products (Wu et al., 2024). Large-scale, long-term clinical trials are needed to confirm their efficacy and safety (Ahmed et al., 2023). Interdisciplinary collaborations among researchers, healthcare professionals, and industry stakeholders will drive innovation and translate scientific findings into practical applications (Bhavnani et al., 2017). Exploring new natural metabolites with anti-aging potential and understanding their interactions with existing therapies will continue to expand the repertoire of effective interventions (Wan et al., 2024).

## 5 Conclusion

The exploration of nutrients and natural products for sustainable drug development against offers significant health benefits. Unlike conventional pharmaceuticals, these natural metabolites often have fewer side effects while maintaining both efficacy and safety. By focusing on the therapeutic potential of metabolites derived from food and natural sources, researchers are uncovering new pathways to enhance health and longevity. Nutrients and natural products have demonstrated their capacity to modulate key biological pathways related to immune function, inflammation, and neuroprotection. Recent studies have highlighted the importance of targeting aging-related processes such as mitochondrial dysfunction, genomic instability, and cellular senescence.

In conclusion, leveraging the potential of nutrients and natural products provides a sustainable, effective approach to aging-related therapies. While this review primarily focuses on vitamins, minerals, and natural products, future research should also consider the role of essential amino acids in aging-related interventions. These metabolites address aging symptoms and target its underlying causes, offering the potential to significantly improve healthspan and quality of life. Anti-aging strategies could be transformed through continued exploration and advancements in delivery technologies and personalized medicine, benefiting individuals and healthcare systems worldwide.
